# Effect of Sit-to-Stand Training Combined with Taping on Spasticity, Strength, Gait Speed and Quality of Life in Patients with Stroke: A Randomized Controlled Trial

**DOI:** 10.3390/life11060511

**Published:** 2021-05-31

**Authors:** Tae-sung In, Jin-hwa Jung, Kyoung-sim Jung, Hwi-young Cho

**Affiliations:** 1 Department of Physical Therapy, Gimcheon University, Gimcheon 39528, Korea; 20160072@gimcheon.ac.kr; 2 Department of Occupational Therapy, Semyung University, Jecheon 27136, Korea; otsalt@semyung.ac.kr; 3 Department of Physical Therapy, College of Health Science, Gachon University, Incheon 21936, Korea

**Keywords:** stroke, sit-to-stand, taping, spasticity

## Abstract

*Background and Objectives:* Spasticity is one of the factors that make it more difficult to control posture in stroke patients. Taping has been used to manage muscle stiffness in various musculoskeletal disorders. Recently, it has been used to decrease spasticity in stroke patients, but the effect of taping combined with therapeutic exercise is still unclear. The purpose of the present study was to determine whether the sit-to-stand (STS) training combined with taping improves the ankle spasticity, muscle strength, gait speed, and quality of life in stroke patients. *Material and Methods:* The study recruited 40 stroke patients, who were randomly divided into two groups: the taping and STS training (TSTS) group (*n* = 20) and the STS group (*n* = 20). The subjects in the TSTS group underwent STS training with Kinesio taping on the tibialis anterior, calf and ankle joint, whereas the subjects in the STS group underwent only STS training. All participants underwent 30 sessions of STS training (30 minutes, 5 days per week for 6 weeks). The present study evaluated the spasticity of ankle plantar flexors by the mean of the composite spasticity score; the muscle strength and gait speed were evaluated using the handheld dynamometer and the 10-meter walk test, respectively, and the quality of life was assessed using the stroke-specific quality of life scale. *Result:* The TSTS group and the STS group showed significant improvements in spasticity, muscle strength, walking speed, and quality of life after the intervention (*p* < 0.05). The level of improvement in the TSTS group was significantly higher in spasticity, muscle strength, and walking speed compared to the STS group (*p* < 0.05). *Conclusions:* The present study demonstrated that STS training is effective for decreasing spasticity in stroke patients and suggested that additional taping intervention further improved this effect. In addition, improvement of muscle strength and gait function was observed with a significant decrease of ankle spasticity.

## 1. Introduction

The sit-to-stand (STS) movement is an essential element in independent, day-to-day life [1], during which, the center of gravity increases and the base surface narrows [2]. Stroke patients are more susceptible to falls and take more time to sit and stand, compared to healthy adults, owing to the impaired posture control [3,4]. Moreover, stroke patients support most of their weight on a healthy side while sitting or standing due to decreased muscle strength and reduced sensory function in the affected lower extremities [5]. STS training method is widely used in the clinic, in order to improve the muscle strength and balance function in stroke patients. Previous studies that employed STS training for the rehabilitation of stroke patients reported significant improvements with regard to the weight distribution [6] and postural sway [7]. Furthermore, Tung et al. reported significant improvements in the posture control and lower limb muscle strength in stroke patients who conducted the STS training [8]. During the STS movement, the distribution of body weight varies in accordance with the position of both the lower limbs. Moreover, Roy et al. reported that the STS training with the affected foot positioned behind the unaffected foot resulted in significant improvement in the weight support pertaining to the affected side in stroke patients [9]. 

However, dorsi-flexion is limited in stroke patients with the spasticity of the plantar flexor, which makes it difficult for the heel of the affected foot to contact the ground during standing and exercise training. Spasticity is a common symptom associated with damage to upper motor neurons, which is a sensory-motor disorder that increases resistance to passive stretch, on account of the hyper-excitability of the stretch reflex [10]. It has been reported that 36–70% of stroke patients experience spasticity [11,12], which is one of the major factors that not only negatively affects function recovery but also degrades the quality of life [13,14]. In addition, a stroke patient with spasticity finds it difficult to control posture, and due to the maintenance of a continuous muscle contraction state, it is difficult to induce proper muscle strength and to perform other movements [15,16,17]. Previous studies reported that spasticity causes morphological changes in muscle thickness in stroke patients [18,19]. Pharmacotherapeutic agents [20], transcutaneous electrical stimulation [21], taping [22], and other therapeutic modalities can be employed to alleviate the intensity of spasticity in stroke patients. Among the aforementioned therapeutic modalities, taping is a method that is not only inexpensive but can be easily applied by anyone without side effects [23]. Additionally, taping could regulate the muscle tone, in order to maintain the balance between the agonist and antagonist muscles during voluntary movement [24]. 

Recently, taping has emerged as a measure to prolong the spasticity relief effect of the Botox injection. In addition, it has been reported to be effective in improving the proprioception and postural control function in stroke patients [25]. A previous study that involved the taping application to the plantar flexor in stroke patients reported that significant improvement was observed in the ankle spasticity and passive dorsi-flexion angle [26].

Although several previous studies have reported the effect of taping on spasticity and muscle strength, most of them have only identified the immediate effects of taping. Although spasticity relief is one of the important issues for the function recovery of stroke patients, the existing treatment methods are focused only on spasticity or apply the same training to all subjects regardless of spasticity. Therefore, the purpose of this study is to confirm the effect of taping on the relief of spasticity during STS training in stroke patients with plantar-flexor spasticity, and also to confirm the effect on the muscle strength and gait function of the lower extremities along with these effects. We applied long-term STS training to the positive control group to verify the anti-spastic effect of exercise training, and in the experimental group, taping was additionally attached along with the same exercise performance as the control group. We hypothesized that spasticity will be reduced after the application of taping. We hypothesized that the additional application of taping to ankle joints would alleviate ankle spasticity more effectively and also effectively improve the muscle strength, gait of the lower extremities, and quality of life in stroke patients.

## 2. Experimental Section

### 2.1. Participants

The present study included 40 stroke patients who were admitted to the K Rehabilitation Hospital in Gyeonggi-do. The selection criteria are stated as follows: stroke patients with unilateral palsy who could communicate independently and walk 10 meters; patients with moderate to severe spasticity (composite spasticity score of 10 or greater) of the affected ankle; and the patients in Stage 3 of the Brunnstrom recovery stages [27]. In this study, subjects with vestibular impairment, sensory loss, and orthopedic diseases affecting sit-to-stand movement were excluded. Among the patients who met the selection criteria, the current study included those who received sufficient information and explanation regarding the study and consented to participate in the same. This study was approved by the Institutional Review Board of the Gachon University (IRB no. 1044396-201805-HR-116-01). The present study employed the G*power3.1.9.2 program to compute the sample size; the sample size was estimated to be 36 subjects (α error: 0.05, force: 0.8, effect size: 0.8625793). Considering the possibility of dropouts, a total of 40 subjects were included in the present study, in accordance with the selection criteria.

### 2.2. Protocol

This study was designed as a double-blinded randomized controlled trial (RCT). This study was conducted from July 2018 to May 2019. Among stroke inpatients admitted to the K rehabilitation hospital, intervention was performed at the hospital for patients who met the selection criteria. 

**Figure 1:**
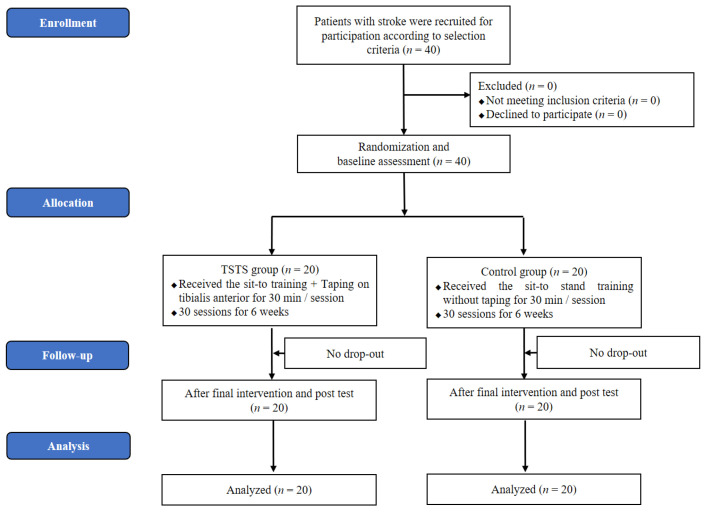
Flow diagram of participants through the study.

In order to minimize the selection bias, the 40 subjects were randomly divided into two groups: the taping and sit-to-stand (TSTS) group and the sit-to-stand (STS) group. For randomization, a number (1 or 2) was chosen from a sealed envelope by a person who did not participate in the study. Thus, 20 subjects were assigned to each group. Subjects in the experimental group underwent sit-to-stand training combined with taping. The training lasted for 30 minutes a day, five times a week for six weeks. Subjects in the control group received sit-to-stand training without taping for the same amount of time. All of the subjects received conventional therapy for an hour a day, five times a week for four weeks. The conventional therapy was patient-specific and consisted mainly of physical therapy and occupational therapy. The current study did not encounter any patient dropout. Thus, a total of 40 subjects were included in the post-training evaluation. The spasticity, muscle strength, walking speed, and quality of life were assessed before and after the training. The participants were evaluated one day before and one day after STS training by three well-trained physical therapists, who were not informed on the subjects and the purpose of this study (Figure 1). 

### 2.3. Intervention

Subjects in both the TSTS and STS groups participated in sit-to-stand training. For sit-to-stand training, subjects were seated in a height-adjustable chair with the hips and knee joints at right angles, and half the length of the thigh bones spanning the chair. The big toe of the paretic foot was placed in the middle of the non-paretic foot. Subsequently, the sit-to-stand movement was performed without arm support. The subjects were allowed to rest for one minute after performing 10 repetitions. The present study used four pieces of straight elastic tape (kinesiology 3NS tape, Golden Health Farm, Korea) with a width of 5 cm for taping therapy. The tape was changed once every three days. The tape was attached to the ankle in a neutral position and applied in four steps. First, in the supine position, the tape was stretched to about 120% of the maximum length, commencing from the middle of the dorsal metatarsal bone to below the head of the fibula. Subsequently, the tape was attached to the tibialis anterior muscle. The second, third, and fourth steps involved the attachment of the tape in the prone position. The second step involved the application of two-pronged tapes. Commencing from the heel, the tapes were attached to the medial and lateral heads of the calf muscles, respectively. In the third step, the tape was attached to the medial and lateral malleoli, commencing from the arch of the sole of the foot. In the fourth step, the tape was attached to both malleoli across the ankle joint [25]. 

### 2.4. Outcome Measurements

The present study used the composite spasticity score (CSS) to assess the spasticity of the plantar-flexor muscles. CSS comprises the assessment of the reflexes of the Achilles tendon, resistance to passive dorsal flexion, and ankle clonus. The Achilles tendon reflex test involves a five-point scale, with higher scores indicating increased reflexes. The passive dorsi-flexion test involves the evaluation of resistance when performing in the maximum range, which is evaluated by doubling the score on the modified Ashworth scale, which is a five-point scale. The scores pertaining to the ankle clonus range from a minimum of one point to a maximum of four points; one point indicates that clonus does not occur, and four points indicate that clonus continues to occur. A total score of less than nine indicates mild spasticity. A score of 10~12 indicates moderate spasticity and a score of 13~16 indicates severe spasticity [28].

A handheld dynamometer was used to test the muscle strength pertaining to the plantar-flexor muscles. In order to assess the muscle strength pertaining to the plantar-flexor muscles, the hip and knee joints were straightened, and plantar-flexion was performed against the handheld dynamometer in a prone position. The handheld dynamometer showed high intra-meter reliability and inter-meter reliability in patients with nervous system injury (*r* = 0.84~0.99) [29].

The 10-meter walk test (10MWT) was used to determine the changes in the walking speed. As a method of measuring the time taken to walk 10 meters, the inter-rater and intra-rater reliability is very high (*r* = 0.89~1.00) [30].

The stroke-specific quality of life (SS-QoL) scale was employed to assess the changes in the quality of life of the subjects. SS-QoL scale is an assessment tool developed in accordance with the characteristics pertaining to stroke patients. The scale comprises 49 items pertaining to a total of 12 domains, including mobility, upper limb function, language function, thinking capability, visual function, self-care, mood, personality, energy, family role, social role, and production capacity. Each question is composed of a five-point scale; the higher the score, the higher the quality of life. Literature has established the reliability of the SS-QoL scale with a Cronbach’s alpha value of 0.80 [31].

### 2.5. Data Analysis

A random number was assigned to the 40 subjects included in the present study. Subsequently, 50% of the subjects were randomly selected using the random sampling method by means of the SPSS program. The selected group was designated as the TSTS group and the remaining subjects were designated as the STS group. The present study used the SPSS version 21.0 to perform the statistical analysis. The normality test of the variables was performed by means of the Shapiro–Wilk test. An independent *t*-test was used to analyze the differences between the two groups and paired *t*-tests were used for intra-group comparisons. The chi-square test was used for the comparison of categorical variables. The statistical significance level pertaining to all the data was less than 0.05. The effect size of TSTS related to STS was calculated using Cohen’s d. 

## 3. Results

### 3.1. General Characteristics of Subjects

In the current study, 40 stroke patients completed the pre- and post-training evaluation. The general characteristics pertaining to the study subjects are shown in Table 1. There were no significant differences between two groups in the spasticity, muscle strength, walking speed, and quality of life at baseline, including general characteristics. Also, no subjects complained of side effects due to taping.

### 3.2. Changes in Spasticity after Training

Both the TSTS and the STS groups showed significant changes in CSS measurement after intervention (*p* < 0.05). In addition, the TSTS group showed more significant improvements in differences between before and after training compared to the STS group (*p* < 0.05; Table 2).

### 3.3. Changes in Muscle Strength After Training

This study observed significant changes with regard to the pre- and post-training muscle strength in both groups (*p* < 0.05). The TSTS group showed a greater improvement with regard to the difference in the muscle strength between pre- and post-training, compared to the STS group, and the difference was observed to be statistically significant (*p* < 0.05; Table 3).

### 3.4. Changes in Walking Speed after Training

Both groups showed a significant improvement in walking speed after the training intervention compared to before (*p* < 0.05). In particular, it was shown that the participants in the TSTS group improved significantly compared to the participants in the TST group in the differences in gait speed between pre- and post-intervention (*p* < 0.05; Table 4).

### 3.5. Changes in Quality of Life After Training

All subjects in both groups participating in the study showed significant improvements in the quality of life after intervention (*p* < 0.05). The TSTS group showed a greater improvement in quality of life than the STS group, but there were no significant differences between the two groups (*p* > 0.05; Table 5). 

## 4. Discussion

The present study investigated the effects of STS training with taping for 6 weeks on spasticity in stroke patients. Accordingly, the TSTS group showed a significant improvement in spasticity compared to the STS group. A previous study that involved the application of taping to the upper extremities of stroke patients reported that the spasticity was significantly reduced compared to the control group, which was attributed to the fact that taping induces self-inhibition of hypertonic muscles, as the muscles are continuously elongated [32]. In a study comparing the effects of casting, taping, and stretching after injection of botulinum toxin type A, it was found that casting and taping had a longer-lasting effect of reducing spasticity and improving range of motion in the ankle joint than stretching, and this result was explained by the difference in application time [26]. In this study, there were no subjects who complained about the side effects of taping, and since it can give stimulation for a long time compared to the treatment applied for a short time, It is believed that taping can be applied easily and simply without side effects to relieve spasticity in stroke patients. In a previous study by Koseoglu, they applied a taping to the tibialis anterior with the performance of a conventional stroke rehabilitation program for 4 weeks and suggested that the spasticity of the lower extremities was completely eliminated in 10 stroke patients [33]. We recruited stroke patients with moderate spasticity, while they mobilized patients with mild spasticity of about 1 with the MAS grade despite being chronic stroke patients. Also, to measure the intensity of spasticity, we used the CSS, and they used the MAS. As the subjects’ degree of spasticity and the measurement methods are different, there are limitations to directly comparing the additional effects of taping between the two studies. However, the results of our study demonstrated that additional taping has a large level of effect size (>0.80), which proves that it is effective for rehabilitation exercise training in stroke patients with moderate spasticity (Table 2). 

In addition, in this study, the strength of the plantar flexor was measured to determine whether the effect of the training could be increased when STS training was performed with taping. As a result, the TSTS group was observed to display a significantly greater improvement compared to the STS group (large effect size: 0.888; Table 3). Similar to our results, Jung et al. suggested that the increase in the range of motion in the ankle joint due to the decrements in spasticity can improve the muscle strength of the affected lower extremity by performing weight support more symmetrically [34]. In this study, it is assumed that the cause of the increase in the muscle strength of the plantar flexor after STS training was due to the increase in the range of ankle dorsi-flexion following the decrease in calf spasticity. In a stroke patient, due to the increase in dorsi-flexion of the affected side, the heel of the foot touches the ground in a sitting position, and thus the correct sensory input of the foot for weight support is transmitted to the brain. In addition, the weight support on the lower limbs of the paralyzed side increases, thereby promoting the activity of the extensor muscles located at the backside of the body. In addition, the increase in sensory input following taping can be estimated as another cause of improvement in muscle strength in the TSTS group. Taping stimulation can influence the enhancement of proprioceptive sensations by promoting more sensorimotor input in rehabilitation training performance [35]. Increased proprioceptive sensation improves motor function by increasing the recruitment of motor units [36]. In addition, the application of taping pulls the muscle belly and increases the overlap of the actin and myosin filament, thereby increasing the possibility of a cross-bridge [37]. 

In past studies, to increase the muscle strength of the ankle dorsi-flexor, botulinum was injected into the plantar flexor to reduce the resistance of dorsi-flexion movement [25,26]. On the other hand, we managed and reduced the degree of spasticity in the plantar flexor itself and increased the extensibility of the muscles, thereby promoting the increase in muscle strength. Our intervention and measurement method is an implementation of a method used for stroke rehabilitation in clinical practice, and through this, we proved that taping intervention, which is additional to exercise training, can be effectively used to improve lower extremity muscle strength in stroke.

The ankle joints and feet adjust balance control against the postural sway caused by internal and external stimuli, absorb shocks, and provide propulsion during walking. In order to perform the aforementioned functions, a sufficient range of motion of the ankle joint, muscle strength, and proprioceptive sensation are required [38]. Interestingly, in our study results, all subjects significantly improved their walking speed after STS training, and this effect was more effectively increased by the application of taping (large effect size: 1.457; Table 4). Various causes may be involved in these results, but we speculate that the increased muscle strength in the plantar flexor is the main cause. A previous study that investigated the variables that affect the walking speed in stroke patients reported that walking speed had a significant correlation with the strength of the plantar-flexor muscles [39]. Moreover, spasticity causes weakness in the muscle strength, limits the range of motion of the joint, and renders proper coordination between the contractions of the agonist and antagonist muscles difficult [11]. In addition, a decrease in ankle spasticity would also have an effect on an increase in walking speed. Spasticity interferes with coordinated movements by the constant contraction of the muscles in the involved joint and also impedes intentional movements. Therefore, it is believed that the anti-spastic effect of taping had a positive effect on the improvement of gait function through this effect.

Huang et al. argued that the limited range of motion due to spasticity causes dysfunction and degrades the quality of life. In this case, it is possible to improve the quality of life through rehabilitation training that reduces the abnormal muscle tone and promotes motor function [32]. However, the present study did not observe any significant difference between the two groups with regard to the changes in the quality of life after training. It is assumed that the effects of training on mental health were inadequate, or the training period was rather too short to affect the quality of life. Furthermore, most of the functional evaluations involved in the assessment of the quality of life include the movements in daily life, such as carrying objects or walking. Consequently, the sensitivity pertaining to the evaluation of the quality of movements attributable to spasticity relief is low.

The present study investigated the effects of STS training combined with taping on ankle spasticity, muscle strength of the lower extremity, gait speed, and quality of life in stroke patients. The results showed that there was a significant improvement with regard to spasticity and walking speed, compared to the STS training without taping. However, owing to the small sample size, the generalization of treatment effects on the basis of the results of the present study is difficult. In addition, both groups performed STS training, but the placebo effect caused by this cannot be excluded because taping was not applied to the control group. Also, it was not confirmed whether the actual proprioceptive sensation or dorsi-flexion angle was changed. Therefore, it is necessary to reconfirm whether the improvement effect is due to the intervention by including a placebo group in future studies, and it is necessary to find out the effect of the intervention method of this study on proprioception and the dorsi-flexion angle. 

## 5. Conclusions

The current study investigated the effects of STS training with taping for 6 weeks on ankle spasticity, lower extremity muscle strength, walking speed, and quality of life in stroke patients. The results of this study suggest that taping may increase the effectiveness of exercise by enabling more accurate sensory inputs during exercise in stroke patients with spasticity. Taping can be applied easily and simply without the burden of time and cost, so it can be effectively used as a rehabilitation aid for stroke patients with spasticity in clinical practice.

## Figures and Tables

**Table 1:** Common and clinical characteristics of the subjects (*n* = 40).

Variables	TSTS Group (*n* = 20)	STS Group (*n* = 20)	*p*
Affected side (Left/Right)	10/10	11/9	0.977 ^a^
Age (years)	56.15 ± 10.39	55.05 ± 9.88	0.514 ^b^
Height (cm)	164.70 ± 9.05	166.45 ± 9.90	0.563
Weight (kg)	61.70 ± 8.66	63.85 ± 8.33	0.429
Duration of stroke (months)	7.05 ± 2.78	6.80 ± 2.50	0.737

Note. Values are expressed as mean ± standard deviation (SD). ^a^ chi-square test; ^b^ independent *t*-test. TSTS group, taping + sit-to-stand training group; STS, sit-to-stand training group.

**Table 2:** The changes in spasticity following intervention.

		TSTS Group	STS Group	*p*	ES
CSS (score)	Pre	12.10 ± 1.89	11.60 ± 1.93	0.413	
	Post	9.15 ± 1.69	11.10 ± 1.71		
	Differences	−2.95 ± 1.39	−0.50 ± 2.67	0.001	1.151
	*p*	< 0.001	0.412		

Note. CSS: composite spasticity score; ES: effect size.

**Table 3:** The changes in muscle strength following intervention.

		TSTS Group	STS Group	*p*	ES
Plantar flexor (kg)	Pre	11.09 ± 2.34	11.51 ± 2.15	0.426	
	Post	15.02 ± 3.42	13.22 ± 1.51		
	Differences	3.93 ± 2.93	1.71 ± 1.98	0.008	0.888
	*p*	< 0.001	0.003		

Note. ES, effect size.

**Table 4:** The changes in walking speed following intervention.

		TSTS Group	STS Group	*p*	ES
10MWT (second)	Pre	25.74 ± 4.62	25.01 ± 4.40	0.678	
	Post	20.11 ± 3.07	23.22 ± 4.65		
	Differences	−5.64 ± 2.89	−1.79 ± 2.37	<0.001	1.457
	*p*	< 0.001	0.004		

Note. 10MWT: 10-meter walk test; ES: effect size.

**Table 5:** The changes in quality of life following intervention.

		TSTS Group	STS Group	*p*	ES
SS-QoL (score)	Pre	155.40 ± 10.95	154.45 ± 10.95	0.785	
	Post	158.40 ± 9.77	156.35 ± 9.98		
	Differences	3.00 ± 3.09	1.90 ± 2.69	0.238	0.380
	*p*	<0.001	0.005		

Note. SS-QoL: Stroke-specific quality of life; ES: effect size.

## Data Availability

Not applicable.
